# Measurement of Orbital Volume after Enucleation and Orbital Implantation

**DOI:** 10.1371/journal.pone.0050333

**Published:** 2012-12-06

**Authors:** Olga Lukats, Tamas Vízkelety, Zsolt Markella, Erika Maka, Maria Kiss, Adrienn Dobai, Peter Bujtár, Attila Szucs, Jozsef Barabas

**Affiliations:** 1 Departmentof Opthalmology, Faculty of Medicine, Semmelweis University, Budapest, Hungary; 2 Department of Oral and Maxillofacial Surgery and Dentistry, Faculty of Dentistry, Semmelweis University, Budapest, Hungary; 3 Kálmán Kandó Faculty of Electrial Engineering, Óbuda University, Budapest, Hungary; 4 Dunaújváros College, Dunaújváros, Hungary; 5 Department of Oral and Maxillofacial Surgery, University Hospitals of Leicester, Leicester, United Kingdom; Semmelweis University, Hungary

## Abstract

**Introduction:**

This article reports experience relating to the measurement of orbital volume by means of cone beam computed tomography (CBCT) and Cranioviewer program software in patients who have undergone enucleation and orbital implantation.

**Patients and Methods:**

CBCT scans were made in 30 cases, 10 of which were later excluded because of various technical problems. The study group therefore consisted of 20 patients (8 men and 12 women). The longest follow-up time was 7 years, and the shortest was 1 year. In all 20 cases, the orbital volume was measured with Cranioviewer orbital program software. Slices were made in the ventrodorsal direction at 4.8 mm intervals in the frontal plane, in both bony orbits (both that containing the orbital implant and the healthy one). Similar measurements were made in 20 patients with various dental problems. CBCT scans were recorded for the facial region of the skull, containing the orbital region. The Cranioviewer program can colour the area of the slices red, and it automatically measures the area in mm.

**Results:**

In 5 of the 20 cases, the first 4 or all 5 slices revealed that the volume of the operated orbit was significantly smaller than that of the healthy orbit, in 12 cases only from 1 to 3 of the slices indicated such a significant difference, and in 3 cases no differences were observed between the orbits. In the control group of patients with various dental problems, there was no significant difference between the two healthy orbits. The accuracy of the volume measurements was assessed statistically by means of the paired samples t-test.

**Summary:**

To date, no appropriate method is avaliable for exact measurement of the bony orbital volume, which would be of particular importance in orbital injury reconstruction. However, the use of CBCT scans and Cranioviewer orbital program software appears to offer a reliable method for the measurement of changes in orbital volume.

## Introduction

The orbits are conical or four-sided pyramidal cavities, which open into the midline of the face and point back into the head. Each consist of a base, an apex and four walls. They protect the eye from mechanical injury.

The base which opens in the face has four borders. The folowing bones take part in their forrnation: superior margin: frontal bone, inferior margin: maxilla and zygomatic, medial margin: frontal, lacrimal and maxilla, lateral margin: zygomatic and frontal. The apex lies near the medial end of superior orbital fissure and contains the optic canal (containing the optic nerve and ophthalmic artery) which communicates with middle cranial fossa. The roof is formed primarily by the orbital plate frontal bone, and also the lesser wing of sphenoid near the apex of the orbit. The orbital surface presents medially by trochlear fossa and laterally by lacrimal fossa.

The floor is formed by the orbital surface of maxilla, the orbital surface of zygomatic bone and the minute orbital process of palatine bone. Medially near the orbital margin, is located the groove for nasolacrimal duct. Near the middle of the floor, located infraorbital groove, which leads to the infraorbital foramen. The floor is separated from the lateral wall by inferior orbital fissure, which connects the orbit to pterygopalatine and infratemporal fossa.

The medial wall is formed primally by the orbital plate of the ethmoid, as well as contributions from the frontal process of maxilla, the lacrimal bone and a small part of the body of the sphenoid.

The lateral wall is formed by the frontal process of zygomatic and more posteriorly by the orbital plate of the greater wing of sphenoid. The bones meet at the zygomaticosphenoid suture.

The adult orbital margin is approximately rectangular with horizontal dimension of 40 mm and a vertical dimension of 35 mm. The length of the medial orbital wall from the anterior lacrimal crest is 45–50 mm, whereas the lateral wall from the rim to the superior orbital fissure is 40 mm. The adult lateral orbital walls are angled 90 degrees from each other or 45 degrees in the anteroposterior direction. The divergent axis of each orbit thus becomes half of 45 degrees or 22.5 degrees.

In the adult human the volume of the orbit is 30 ml. Because of the complicated anatomical structure of the bony orbit, measurement of its volume is difficult. The problems are mainly due to the irregular inner border, holes and fissures. We have now made use of Cranioviewer orbital program software [developed by Vizkelety and Markella], together with cone beam computed tomography (CBCT), to investigate both orbits in 20 patients with intraorbital implants and in 20 patients with various dental problems (otherwise healthy). To the best of our knowledge, there have been no reports in the literature of using CBCT scans for orbital volume measurement. Merely a few literature articles mention an orbital volume decrease after enucleation in adults. Our primary aim was to establish whether there is any detectable difference between the volumes of the healthy and the operated orbits, or between those of the healthy left and right orbits.

## Patients and Methods

The study received ethical approval from Semmelweis University, Budapest, and complied fully with institutional ethical protocols and the guidelines of the Helsinki Declaration. A large-volume CBCT scanner (iCat Classic, Xoran Technologies, Ann Arber, Michigan, USA) was used, with the following parameters: 120 kV, pixel size 0,3 mm, slice increment 0.3, and FOV height 6 cm. This type of CBCT can image not only the facial skull, but also the orbital cavity. CBCT scans were made in an attempt to detect changes within the orbital implants in vivo in 30 patients. At the time of the scan the position of the head in the CBCT was close to the Fankfurt horizontal. The gained selection of data may be rotated freely. In ten cases the situation of the heads was not so good, as can be accepted, for this reason we cannot rotate them properly for measurement. These cases were excluded from the patients. We used only that cases whose heads were in the desired position.

The scans utilized for orbital measurements in this study were from 20 of these subjects (8 men, 12 women; mean age 43.85 years, range 14–76). Enucleation had been peformed because of severe injury in 9 cases, a painful eye in 5 cases, an intraocular malignant melanoma in 5 cases, and retinoblastoma in 1 case. FCI synthetic hydroxyapatite had been used as the orbital implant for volume replacement in 12 cases, and aluminium (Bioceramic) in 8 cases. In 18 cases the implantation had been primary, and in 2 cases secondary. The diameter of the implant was 20 mm in 12 cases, and 18 mm in 8 cases.

**Figure 1:**
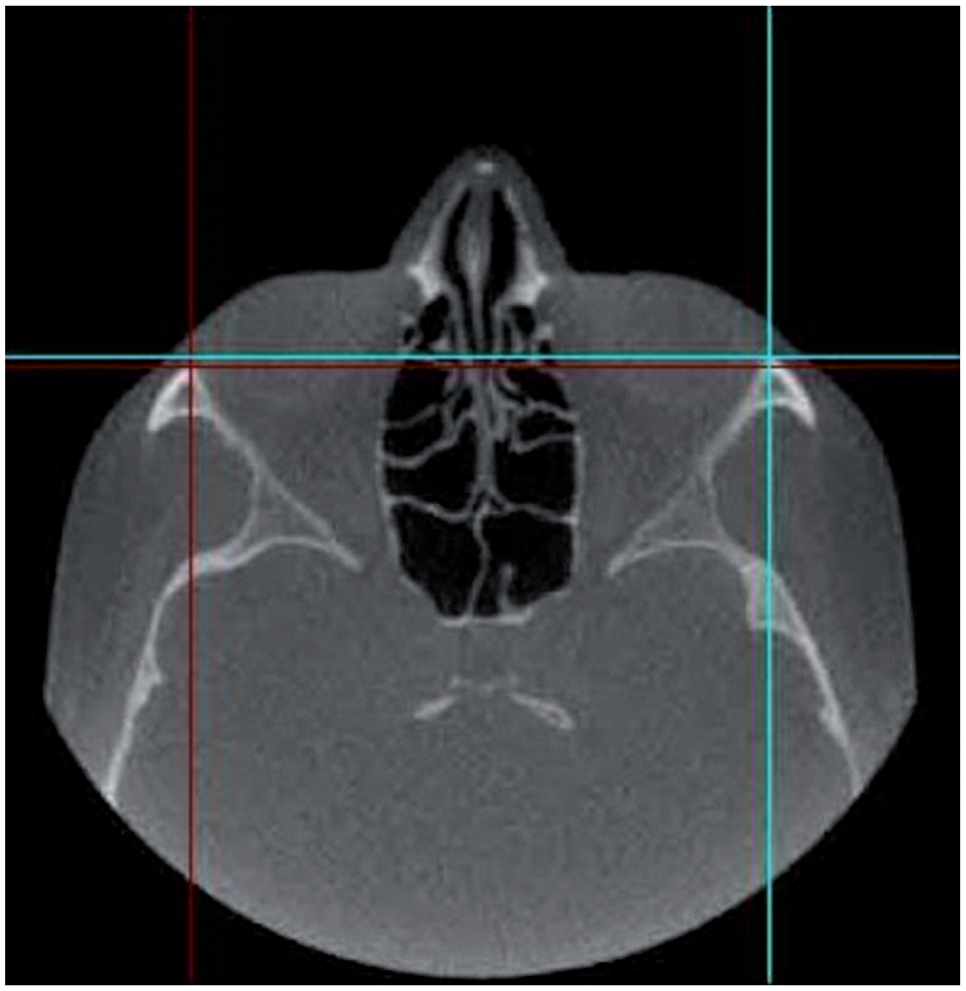
The orbital frame is denoted by red and blue lines in the axial scan.

**Figure 2:**
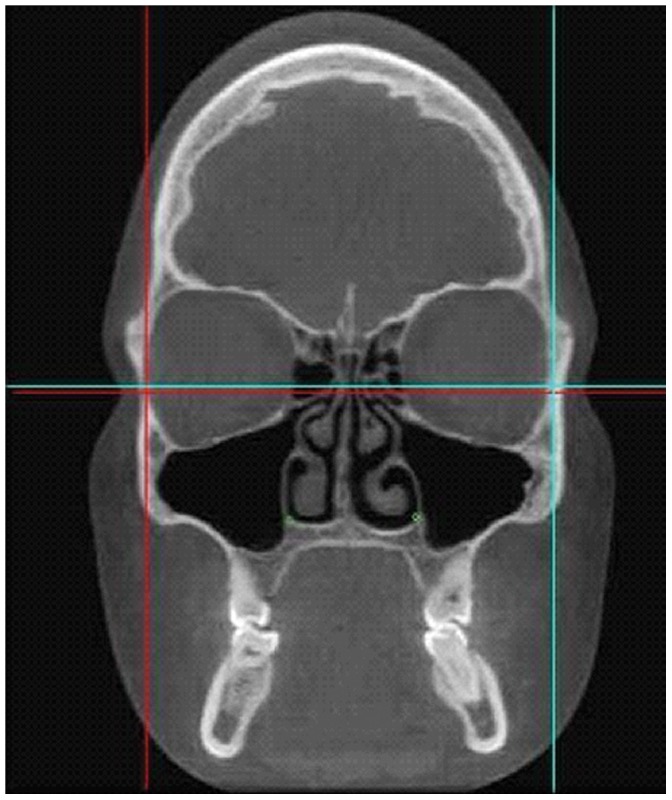
The bony orbital frame is denoted by red and blue lines in the coronal scan.

**Figure 3:**
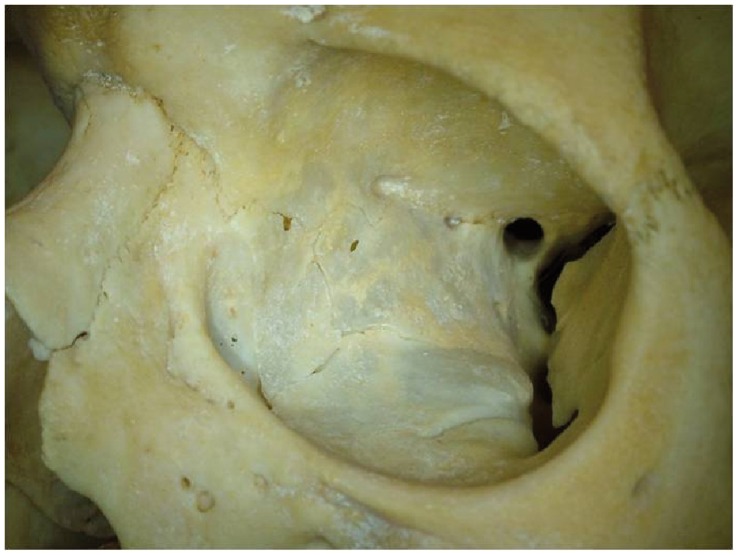
Normal orbital bony cavity with holes and fissures.

**Figure 4:**
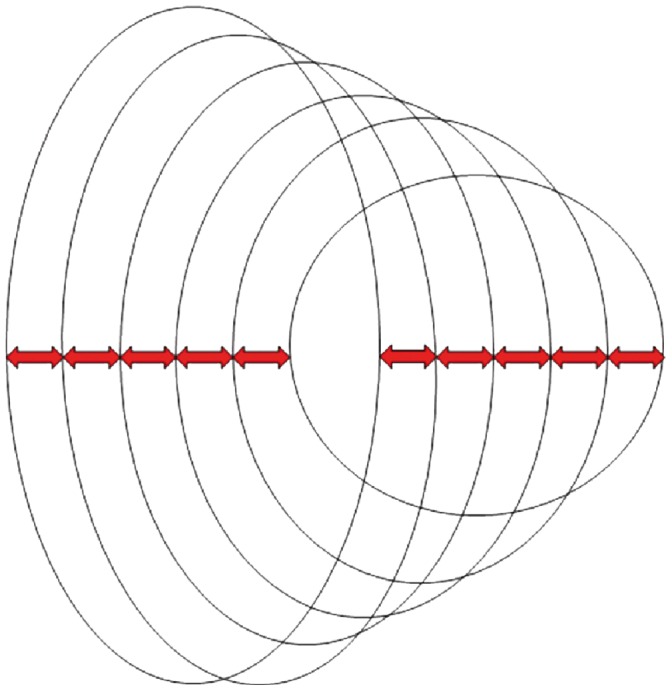
Slices were made at 4.8 mm intervals in the ventrodorsal direction in the frontal plane. Five slices could be made from the orbital frame to the apex.

**Figure 5:**
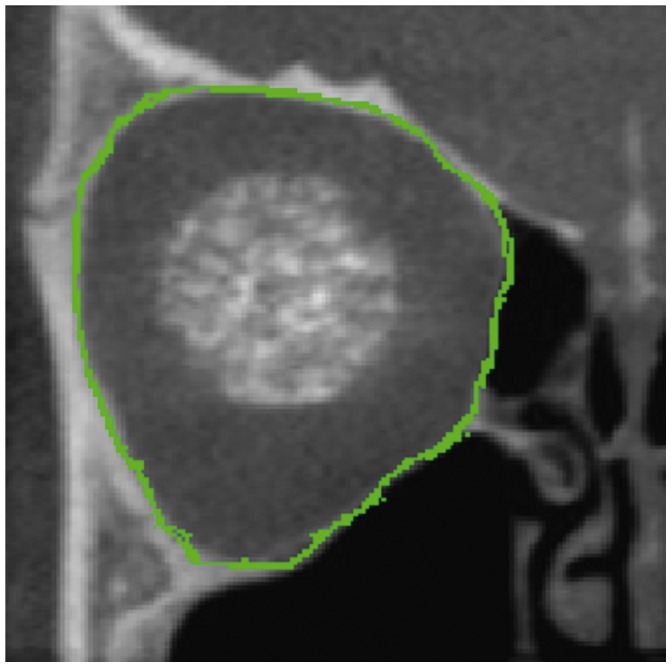
The bony orbital border is denoted by a green line.

**Figure 6:**
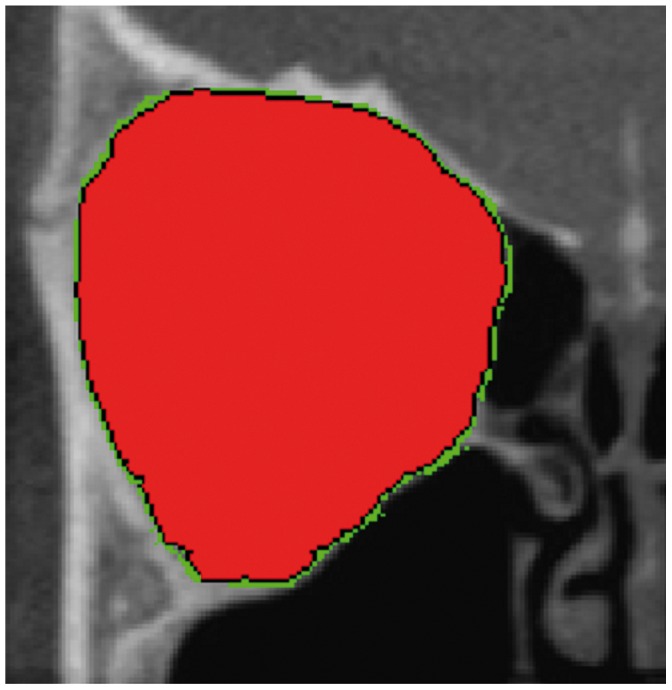
The program automatically colours the area red and measures it in mm^2^.

The Cranioviewer orbital program software was used in conjuction with the CBCT scans for special volume measurement. We set the image in a way that the most dorsal points of the right and the left orbit entry may be connected with a straight line, which at the same time is the level where the most ventral points of the orbit constitute a closed circle. We set the head in vertical position at the time of scanning, then the analogue images of the orbit get into nearly identical levels. At the same time in the skull we may present and measure the area of the frontal image of both the healthy and the implanted orbit and the volume of the orbit once we know the distance between the different levels. The orbital frame can be denoted by red and blue lines in axial [Fig. 1] and coronal [Fig. 2] scans. Slices were made at 4.8 mm intervals in the ventrodorsal direction in the frontal plane. Five slices could be made from the orbital frame to the apex [Fig: 3–4] The program can outline the bony border and enclose the foramens and fissures with a green line [Fig.5], fill every slice with red colour [Fig.6], and measure the area of the slice in mm^2^.

The shortest follow-up time (between enucleation and CBCT examination) was 1 year, and the longest was 7 years.

Results were derived from measurements by 3 different persons, each of whom made each measurement 3 times. In all patients, both orbital cavities were measured with this technique. The paired t test was used to compare group differences. P values < 0.05 were considered to be statistically significant.

## Results: [Table: 1]

**Figure 7:**
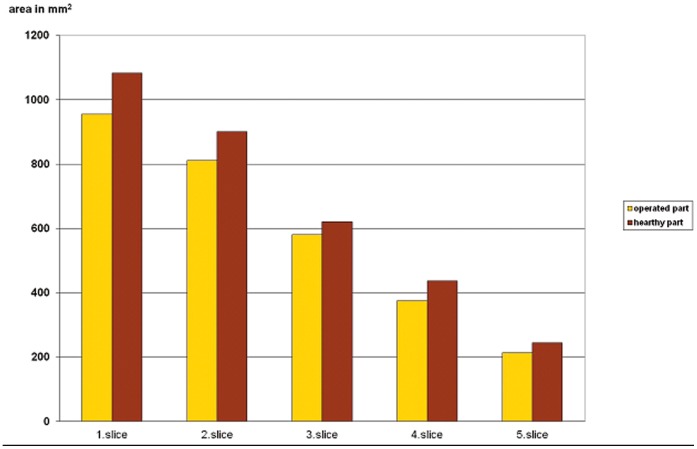
Data on patient No 1, demonstrating significant differences in the measured slices between the operated and the healthy orbit.

In 5 patients, there was a significantly lower volume for the first 4 or all 5 of the measured slices in the orbit containing the implant than in the patient’s own healthy orbit. [Fig. 7] [In patient No. 4, one of the 2 secondary implantation cases, enucleation had been performed in early childhood because of retinoblastoma. In spite of the fact that her implantation had been carried out in adulthood, she proved to be an example of a volume reduction after childhood enucleation. Patient No.5 was 14, and patient No.1 was 16 years old at the time of enucleation and primary orbital implantation. (At these ages, the bony orbita is fully developed and these patients therefore deserve inclusion in the study group). In 12 patients, the measured value was significantly lower in the operated orbit in 1,2 or 3 slices In 3 patients, there were no significant differences between the slices in the operated and the healthy orbits.

**Table 1:** Data on the study group patients.

case	Gender-age	follow uptime - year	significant difference in slices				
			1	2	3	4	5
1	male (16)	4	**yes**	**yes**	**yes**	**yes**	**yes**
2	female (66)	7	**yes**	**yes**	**yes**	**yes**	no
3	female (57)	3	**yes**	**yes**	**yes**	**yes**	**yes**
4	female (26)sec	1	**yes**	**yes**	**yes**	**yes**	**yes**
5	female (14)	4	**yes**	**yes**	**yes**	**yes**	no
6	female (46)	2	no	no	no	no	no
7	female (419	4	no	no	no	no	no
8	female (14)	6	no	no	no	no	no
9	male (20)	6	no	**yes**	**yes**	no	**yes**
10	male (43)	5	no	no	**yes**	**yes**	**yes**
11	female (48)	4	no	no	no	**yes**	no
12	male (76)	2	no	no	no	**yes**	**yes**
13	male (59)	1	no	no	no	**yes**	**yes**
14	female (35)	3	no	**yes**	**yes**	no	**yes**
15	female (50) sec	4	**yes**	no	no	**yes**	**yes**
16	male (35)	3	no	no	**yes**	no	no
17	male (35)	2	no	**yes**	no	**yes**	**yes**
18	female (65)	1	no	no	no	**yes**	no
19	female (38)	4	no	**yes**	no	**yes**	no
20	male (60)	7	no	**yes**	**yes**	no	**yes**

**Figure 8:**
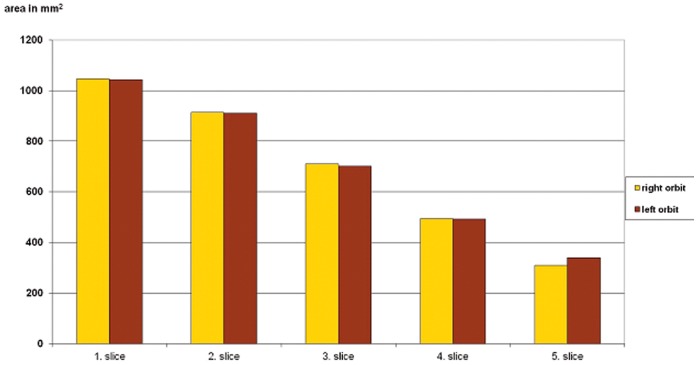
Data on the control group, demonstrating no significant difference in the measured slices between the right and left orbits.

The control group, which consisted of 20 patients, who had never suffered any orbital injury or undergone any orbital opearation, exhibited a normal skeletal skull shape without any facial asymmetry. Their CBCT scans were made because of dental problems. In this group, no significant differences were measured between the right and left orbital cavities [Fig. 8].

## Discussion

Multislice CT and MRI allow multiplanar imaging of both the normal and pathological anatomy. In the CT technique, the scan plane is planned from a lateral scout to be parallel to the infraorbital-meatal line approximating the orbital nerve plane. Slices 3 mm thick are preferred for routine soft tissue visualization on spiral CT because of the increased noise with thinner slices. 3D surface-shaded models may by produced when craniofacial surgery is planned, providing an adequent demonstration of the bone anatomy in the context of fractures of the orbit and craniofacial abnormalities [1].

With CT volume measurements, the task of CT scanning is changed from creating images to determining the number of pixels belonging in a predefined density range. Region-growing algorithms have been used to determine the number of pixels of each category for each slice. When multipled by a conversion factor, the summation of all pixels for each category represents the estimate of the total. Although there are variations among individuals, the range of normal adult measurements generally lies within 2 SDs of the mean. The bony and soft-tissue volumes are slightly greater in men as a group than in women. There are small differences in men as a group compared with women, but minimal differences between the right and left orbits in the same person [4].

Ji et al. [8] examined morphologic orbit parameters of Chinese adults. For example, the bony orbital volume, orbital foramen area and orbital rim perimeter were measured on 3D models through the use of a 3D reconstruction technique.

The differences between the two orbits and between the two sexes were analysed. To ensure accuracy, manual segmentation was used to define the boundary of the orbit. The borders of the whole orbital content were defined by the four orbital walls. The posterior boundary was the junction of the medial and lateral walls at the optic foramen. A simulated surface was determined that covered the orbital foramen with the orbital rim. This surface was used to separate the whole orbital content into 2 parts, the posterior part being defined as the bony orbit. In this way, the area of the orbital foramen and the perimeter of the orbital rim could be calculated automatically. The measurement method demonstrated the high reproducibility of the results. The orbital volume and other anatomic parameters indicated that the two orbits were symmetric. The orbital size proved to be significantly smaller in women than in men, but in a given individual there was no difference between the two orbits.

Orbital blowout fractures are among the injuries most commonly observed in the facial region. One of the most important aspects in the reconstruction of orbital wall fractures is restoration of the normal orbital volume. Accurate preoperative measurement of the orbital volume is invaluable in predicting and (if it leads to operative intervention) preventing post-injury enophthalmos which is a common complication, optimum reconstruction of which also demands an accurate estimation of the orbital implant volume [11].

Thirty facial CT scans were utilized to measure 30 normal orbits by using an image analysis program (Dextroscope, Singapore). Calculation of the orbital volume from coronal scans underestimates the volume as compared with axial scans, and the criterion for the anterior limit of the measurement can affect the volume determination. Three novel cephalometric angles that can be obtained by 3D image analysis with stereoscopy may account for the inaccuracies seen on coronal scans [10].

In 3 series of young, growing rabbits, varying amounts of intraorbital tissue were removed from the right orbit by evisceration, enucleation and excenteration. The left orbit was maintained intact as a control for the operated right side. A removable permanent elastic rubber-based imprint of the clean orbit was made. The volume was calculated from the weight and specific gravity of the orbital imprint. After the excision of orbital tissue, the orbit continued to increase in size, but at a slower rate than that for the unoperated orbit. In general, there was a direct correlation between the lack of intraorbital mass and the decrease in orbital growth [12].

Koppel et al. examined 5 dried skulls with prosthetic globes and periorbita by non-helical scanning with an Elscint 2400 CT scanner. The images obtained were processed with the Analyze software package and the results were compared with the volume of the intraorbital prosthesis determined by a volume-displacement gravimetric method. The Analyze software in combination with the non-helical CT scanning and the protocols used for the automated measurement of orbital volume in that study did not prove sufficiently accurate for clinical application [9].

Fitzgerald et al. present a method that assesses globe movement following monobloc distraction, using computed tomographic scan data. A key indicator for monobloc distraction is globe subluxation, and in the study a quantitative assessment of globe movement was performed and compared it with bone movement. Using the three-dimensional reconstructed images from CT scan in the three-dimensional voxel imaging software, a single operator identified 10 landmarks, 8 on the surface of the skull and 2 within the center of the globes. In all 10 patients measured, left and right globes were moved anteriorly as a result of the osseous distraction. This is the first study published that quntitatively assasses the three-dimensional movement of the globes following craniofacial surgery [4].

Nout et al. examined the influence of Le Fort III advancement on orbita volume, position of the infra-orbital rim and globe in patients with syndromic craniosynostosis suffering from shallow orbits due to midface hypoplasia. CT-scans were carried out in a supine position using the same scanner and had a slice thickness of 1,25 mm. The MevisLab software program was used to import and analyze the CT-scans. In all slices, the anterior boundary was defined as a straight line from the most antero-superior point of the infra-orbital rim to the most antero-inferior point of the supra-orbital rim. When there were bony interruptions (orbital foramina, fissures) a perpendicular straight line was drown between the nearest bony boundaries. After LF III advancement, the orbital volume increased significantly. There was no statistically significant difference between the preaoperative and postoperative left and right orbital volume. Since the anatomical boundaries of the bony orbit are complex, manual segmentation of datasets is necessary. There have been no reports concerning the evaluation of infra-orbital rim and globe position after LF III advancement using a 3D CT method. Their study demonstrates a significant orbital volume gain and anterior movement of the infra-orbital rim following LF III advancement. The position of the globe was relatively unchanged [13].

CBCT has a number of advantages relative to conventional CT. The i-CAT™ cone beam provides unprecedented imaging of the maxillofacial area with less radiation than with traditional fan beam CT systems. The specific advantages of the i-CAT include:

- The open format: The head of the patient is stabilized through use of a chin rest, and the X-ray unit and detector rotate around the head. In this way, the patient does not experience a feeling of claustrophobia.
- The rapid scanning: The rotational scan requires no more than 20 seconds, and the entire procedure less than 5 minutes.
- The comfortable positioning: The positioning is more convenient with the patient seated rather than lying down.
- The detail attained: The maximum image resolution [0.25 mm] provides exceptional bony detail.
- The low dose: The i-CAT utilizes the same type of low-energy dental X-ray source as in existing panoramic dental imaging systems, which results in an effective exposure dose similar to that for a dental perinpical full-mouth X-ray series or a combination of panoramic and bitewing radiographs.
- The full 3D volumetric scan: Multiple images we generated in various planes, which allows a multidimensional assessment.
- The panoramic images: Panoramic images can be generated and customized to fit the jaw of the individual patient. Such images are very familiar to dentists, but, unlike radiographic film panoramic images, the i-CAT panoramic images are distortion-free (Med. Net.), [2], [3].

The study by Howard et al. [7] led them to conclude that enucleation of one eye in infancy or childhood, accompanied by the insertion of an implant, does not cause any cosmetically significant orbital variation.

The postnatal bony orbital development in humans is characterized by rapid growth in the first 3 years, during which ≈50% of the development occurs. Approximately the same amount of growth takes place during the following 10 years [5].

Twenty-nine patients with acquired anophthalmia were examined clinically and with high-resolution CT; 8 of them had been enucleated in early childhood, at ages between 0.4 and 8 years, and 21 in adulthood, at ages between 15 and 53 years. The bony orbital volume was reduced in all these patients with long-standing anophthalmia, e.g. by 3.8% 7-13 years after enucleation in those enucleated during adulthood. The date provided strong evidence that, in both childhood and adults, enucleation is associated with a reduction of the bony orbital volume, and that the reduction is associated with the progression of time. Clinically, none of the patients displayed signs of facial asymmetry. There was no difference between patients with or without primary orbital implants, suggesting that the post-enucleation bony orbital development in humans is not influenced by implantation. The adult bony orbit has been regarded as a stable compartment not affected by enucleation. Some early observations suggested that enucleation might induce shringkage of the bony orbit, but this has not yet been proved [6].

### Conclusions

Whereas no significant difference was observed between the two orbits in 20 subjects who had not undergone enucleation and implantation, a similar result was found in only 3 of the 20 patients in our study group. In the remaining 17 patients, significant differences between the operated and the healthy orbit were observed in from 1 to all 5 slices. Further investigations are necessary to establish what structural changes are responsible for this volume reduction. It is to be hoped that analogous studies will lead to the development of computer software specificially designed to measure the volume on CT scans more accurately and reproducibly, which would be of great value in estimations of orbital changes after enucleation, in calculations of the diameter of the orbital implant to be used for volume replacement and in injury cases when appropriate orbital wall and volume reconstruction is of high importance.

The adventage of the software we apply: measuring the area of the cross-sections at every 4,8 mm stepping in ventrodorsal direction has the advantage that we gain information also about the ventrodorsal localization of the orbit deformation and not only about the shrinking or swelling related to the volume of the whole orbit. So this ventrodorsal registration is best suited for the examitation of the anophthalmic orbit.

In the course of our investigation it became evident, that the comparison of the area of the analogue frontal levels gives a much clearer picture of the changes in the morphology of the bony orbit, than evaluating the orbits as an unified volume.
